# 

**Published:** 1890-05-31

**Authors:** 


					L
wr?1
Nn i?wc' VilNE
ii??, Vol. vm.-
-May 31st, 1890.
Mistered' Ui< VJLiJ" m3,J 0iSl? i0^u-
tt4ag^fo^; ^.e General Post Office as a Newspaper for
ssioa in the United Kingdom and Abroad.]
WITH EXTRA NURSING SUPPLEMENT.
Fer Annum, 6s. 6d.j post free.
Monthly Farts, 6<L; post free 8d,
Ditto per Annum, 8s.. post free.
A WEEKLY JOURNAL OP
Science, jflheMchte, IRursiitg anir {f^IjilantijropS*

				

